# Deep-Learning-Based Segmentation of Fresh or Young Concrete Sections from Images of Construction Sites

**DOI:** 10.3390/ma14216311

**Published:** 2021-10-22

**Authors:** Woldeamanuel Minwuye Mesfin, Soojin Cho, Jeongmin Lee, Hyeong-Ki Kim, Taehoon Kim

**Affiliations:** 1 Department of Architectural Engineering, Chosun University, Gwangju 61452, Korea; minwuyemesfin35@gmail.com (W.M.M.); thoonkim@chosun.ac.kr (T.K.); 2 Department of Civil Engineering, University of Seoul, Seoul 02504, Korea; soojin@uos.ac.kr (S.C.); n2atnut@uos.ac.kr (J.L.)

**Keywords:** concrete, section, construction site, deep learning, convolutional neural network (CNN), image segmentation

## Abstract

The objective of this study is to evaluate the feasibility of deep-learning-based segmentation of the area covered by fresh and young concrete in the images of construction sites. The RGB images of construction sites under various actual situations were used as an input into several types of convolutional neural network (CNN)–based segmentation models, which were trained using training image sets. Various ranges of threshold values were applied for the classification, and their accuracy and recall capacity were quantified. The trained models could segment the concrete area overall although they were not able to judge the difference between concrete of different ages as professionals can. By increasing the threshold values for the softmax classifier, the cases of incorrect prediction as concrete became almost zero, while some areas of concrete became segmented as not concrete.

## 1. Introduction

Visual inspection is an important nondestructive way to monitor the condition of concrete structures. Conventionally, experts visit the construction sites or existing structures to observe the condition of the concrete and take pictures or videos, i.e., a manual inspection. Through the inspection, information including the surface condition, location and depth of cracks could be investigated, and, by very experienced experts, even the moisture condition and the binder composition of concrete can be inspected indirectly. Meanwhile, automated monitoring techniques, especially various computer vision solutions have been proposed to solve the inconvenience of the manual inspection.

The implementation of computer-based image processing methods for concrete structures and materials progressed gradually from filtering- and thresholding-based techniques, such as histogram analysis and brightness adjustment, to artificial-intelligence-based techniques. A number of studies have been conducted on the filtering and thresholding techniques to process images of concrete, many of which are related to understandable pattern recognition and object localization such as crack detection and surface assessment. Joshi et al. [1] investigated the classification of different mixtures of concrete and their composition from X-ray computed-tomography (CT) images using a thresholding algorithm. Hoang et al. [2] identified and calculated the crack width and length from RGB images by using a thresholding technique, called Min–Max gray level discrimination (M2GLD), which could adjust the image’s intensity to increase the crack-detection results. Kabir et al. [3] implemented gradient-based image processing techniques and edge detectors to assess damage in concrete structures from captured images. Lee et al. [4] and Rivera et al. [5] also used different morphological feature extraction and binarization mechanisms to detect cracks on concrete walls. Choi et al. [6] also identified phenolphthalein-sprayed carbonation regions of concrete using morphological and thresholding techniques, which were found to be very effective in detecting the areas accurately. Most of the research done was related to the detection of discontinuities and failure mechanisms which are, from the standpoint of pattern recognition, not difficult and do not insist on an image database. Even as a whole, pattern recognition is an ‘ill-posed problem’, but objectified image segmentation is a well-defined technique to implement, which will be very crucial to the proposed study [7,8].

Recently, deep learning (DL)–based techniques in the construction industry are becoming very popular due to a number of deep neural network architectures following AlexNet [9] and training techniques for the deep architectures. Convolutional neural networks (CNNs) are one of the subdivisions of DL initially developed for optical character recognition [10] and expanded to image and video processing. One of the tasks CNNs handle well is semantic segmentation that infers labels for every pixel in an image to predict the shapes of objects or regions. Recently, various semantic segmentation models have been proposed [11,12] such as SegNet [13], U-Net [14], and DeepLabV3+ [15]. Due to their large number of parameters and functions, these models resulted in an accuracy closer to that of human beings [16,17,18]. 

In 1993, a paper was published that investigated a neural-network-based crack-detection technique on pavements [19], which is the first known application of a neural network model to image recognition in the field of civil engineering, to the best of the authors’ knowledge. The result was very promising with some need for minor adjustments. Since then, different studies were conducted that are useful for the monitoring and maintenance of civil structures, especially concrete structures. Kim and Cho [20] detected and quantified different type of cracks on a concrete wall by using AlexNet CNN architecture. They also investigated a method called mask and region-based CNN models on crack detection the following year [21]. Song et al. [22] used a CNN model to segment concrete images to determine the content of air voids in the concrete mix. The above-mentioned studies and others related to the image-based classification research have focused on the identification of specific defects on concrete structures such as crack damage, efflorescence, and other related deteriorations and damages. 

On the other hand, in this research, the image segmentation of concrete section and area was attempted. Segmentation in various civil structures such as roads, pavements, concrete walls, or guardrails from the RGB images has been studied in the aspect of computer vision for autonomous vehicles [9,10,11,12]; however, there has been no attempt at performing segmentation on concrete construction sites [23]. The automatic separation of the concrete section from the complex images from the construction sites or existing structures will be highly beneficial to monitor and maintain the region of interest on concrete structures both under construction and during operation. 

Through the course of the development of deep-learning-based techniques, various types of CNN models were proposed. These models had their own unique structures and characteristics that were only suitable for certain tasks. Some were more extensive than others, and some possessed a broader set of parameters than others. As a result, it was important to examine and select a model that was compatible with the research objectives. In this work, the CNN architectures were used to classify the area covered by fresh or young concrete at pixel level from the RGB images taken from construction sites under various conditions. A comparison between the performances of various architectures was conducted through different statistical metrics as well as a qualitative evaluation on the segmented area. The main contribution of this work focuses on the tendency of these architectures and their implementation on a database composed of a series of images, which will be able to objectively determine what is concrete or what is not.

## 2. Investigational Methodology

This section contains the procedures implemented on the CNNs for concrete area detection. This study concentrated on the procedures of acquiring the dataset, the steps involved in the development of the labeling method, and finally on finding the most appropriate pretrained CNN model that can be applicable for various purposes. The entire procedure was divided into three basic steps: data acquisition, data training, and data evaluation, which are detailed below.

### 2.1. Data Acquisition

The dataset is an image directory where the images are labeled with their respective classes. Classes are the variables or categories that are on the images that the CNN is going to classify. The classification is somehow similar to binary classification in which, the output can have only two values, a negative class or a positive class. However, it is also equipped with a probabilistic score that gives the probabilistic values of each pixel or object in the image for both classes. Since our objective was to determine the area covered by concrete, the first class was called ‘concrete’ and the section that is not concrete as a whole was considered as another class and called ‘nonconcrete’.

The dataset was a cluster that contained 1059 images taken at different construction sites in South Korea with a conventional digital camera. The images contained different phases of construction stages from the formwork preparation, rebar placement, concrete placement, and curing. They also contained different situations related to concrete construction and placing, which will be briefly discussed in the next section. These images were organized randomly and labeled to avoid bias. The images were gathered with different resolutions, which then were resized to 224 × 224 or 227 × 227 pixel resolutions, according to the network input size.

After the resolution adjustment, the images were labeled at the pixel level. Note that the regions of interest (ROI) in the images can be labeled in two ways, named rectangle (or polygon) labeling and pixel labeling. The rectangle or polygon labeling is usually used to label discrete and distinct objects that have definitive patterns and edges that are easily recognizable and teachable. Cars, person’s face, and any objects that have a definite shape are examples that can be labeled by object or line labeling. These labeling methods are very crucial in object detection and localization techniques, which are applicable in pattern recognition [24]. On the other hand, a pixel-to-pixel labeling method is more appropriate for this research since the area covered by concrete does not have a lot of recognizable patterns or distinctive shapes other than the color [25].

**Figure 1:**
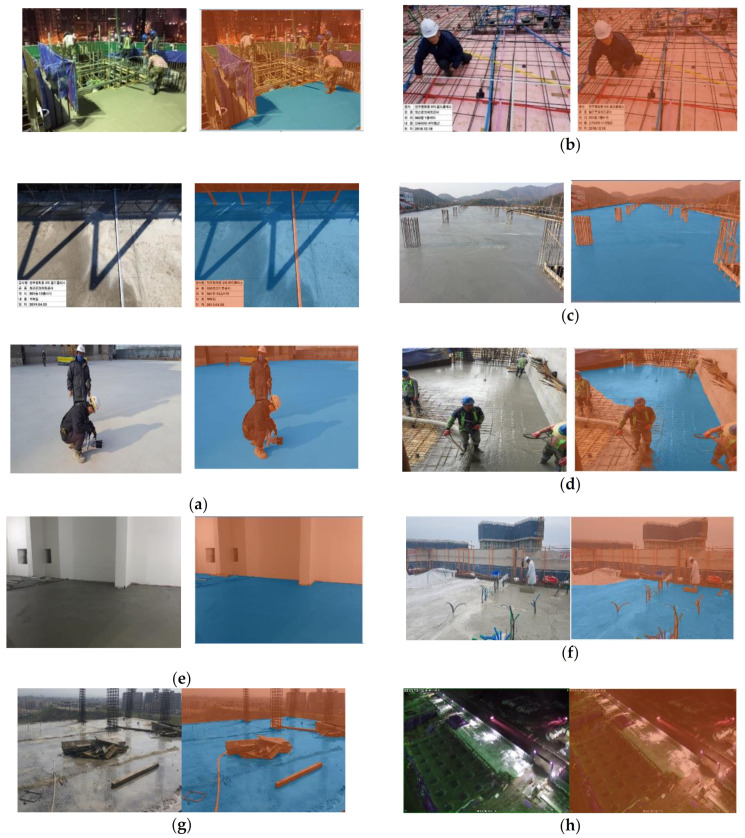
Examples of criteria of pixel-based labeling for construction site images (original: left; labeled: right; red: nonconcrete; blue: concrete): (**a**) Every concrete area was labeled, even the shadows and the dark spots. (**b**) The whole image was labeled as ‘not concrete’ since there were no traces of concrete. (**c**) The reinforcement bars were avoided as much as possible. (**d**) The people were omitted. Moreover, the fresh concrete below reinforcements was also omitted. (**e**) Only the fresh and young concrete was labeled as concrete. Old concrete and concrete covered by paint was omitted. (**f**) When the concrete was covered with a transparent sheet, the areas where the sheet was in contact with concrete were labeled as ‘concrete’. (**g**) Wet regions or concrete covered with water was also labeled as ‘concrete’. (**h**) When the area covered with concrete was in an extreme darkness, it was labeled as ‘nonconcrete’.

The labeling was done according to the example shown in Figure 1. As mentioned above, different images from different situations were taken. Based on that, different adjustments were done in the labeling process to find the possible classification of images. It should be mentioned that the concrete in the construction sites could be classified into three categories: fresh concrete before setting, young concrete after setting but needing a proper curing process, and old concrete requiring no additional curing but needing long-term maintenance. 

For the construction site management, fresh and young concrete should be monitored as they need appropriate curing processes such as moderated temperature and humidity. In this study, fresh and young concrete were labeled as ‘concrete’ as shown in Figure 1, while the old one was not. In the future works, if necessary, the fresh, young, and old concrete could be labeled (or clustered) separately.

After the labeling, the data were separated into two sets, containing 80% (about 847 photos) of the data in the training set and the remaining 20% (about 212 photos) in the testing set. The separation was randomly performed to avoid a biased training dataset. Labeling is a very crucial step as mentioned above. In supervised learning, solid and tangible true data are important since the validity of the result depends on it. Labeling the images provides a ground truth (gTruth) of the data. The gTruth is the true value of the dataset, which stores the correct value and spatial orientation of labeled classes that are going to be used for the training and evaluation process. It is important that the gTruth has to be properly generated since it is the foundation of the training process. It is also relevant in the performance evaluation of the testing images, which is in turn valuable for the validity of the trained model. 

### 2.2. Data Training

**Figure 2:**
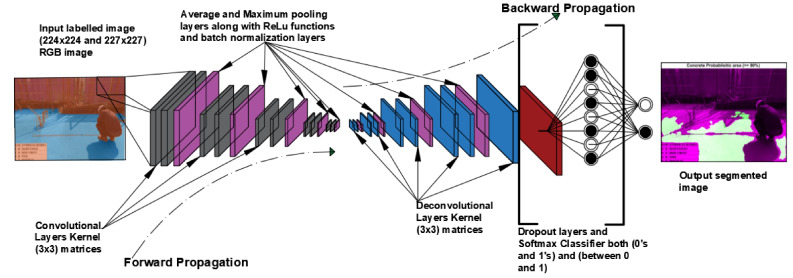
General architecture of CNN model for present work (modified from [17,29,30]).

Training a deep neural network from scratch without pretrained datasets, i.e., unsupervised learning, is an all-embracing work, and it will not be practicable since it requires a very large number of datasets. Therefore, usually a pretrained CNN architecture is used as mentioned in the above explanation, i.e., transfer learning. Transfer learning has been the main beneficiary in the current deep-learning-based segmentation works. Transfer learning is extracting knowledge from previously classified tasks and applying that knowledge to the targeted classes [26]. It provides an effective method of training a large network using scarce training data without overfitting [27]. This is important in boosting the efficiency of the training time and avoiding unnecessary architecture development from the ground up. Therefore, in this investigation, three DL segmentation models with pretrained CNN backbone architectures were used. DeepLab V3+ was used as a one of the state-of-the-art segmentation models, whereas ResNet 18 and 50 [16] were used as base encoder networks. SegNet was the other model that was implemented for the segmentation process using VGG 16 [28] as a backbone encoder. A fully convolutional network (FCN) model using AlexNet [9] was used after remodeling the layers and optimization parameters. As a result, data training was done by tuning their parameters and features. Although the detailed architectures of these models are different from each other, the general architecture of the models is somehow similar as presented in Figure 2.

Each of the CNN models followed more or less the same procedures in the training process, starting from the usage of the same dataset to the testing process. The detailed characteristics and parameters of the CNN models are presented in Table 1.

Data augmentation techniques were implemented to create a different dataset for every training set. Geometric transformations, one of the types of data augmentation methods, was used for this study. All the training, datasets were translated in both the *x* and *y* directions to a specific degree every time the images were trained. This was helpful in circumventing overfitted outcomes every time the models were trained. It also prevents the network from memorizing the exact details of the training dataset. 

After the gTruth dataset was generated and all these conditions were set, training was conducted using the training parameters that are stated below in Table 2. 

The manipulation of mini-batch size and epoch is of a great importance due to the fact that they influence the accuracy of the model. They are also closely related to the other parameters especially the learning rate. The main reason behind this is the effect of the generalization gap (over fitting and underfitting). A larger batch size and epoch does not imply it will result the higher accuracy of results, thereby increasing the generalization gap. In most situations, a lower batch size results in a better output [31]. There is also a GPU requirement when dealing with these hyperparameters. Therefore, proper and efficient values of mini-batch size and epoch should be found in terms of accuracy and execution time. The training execution environment is also described in Table 3. 

After the above-mentioned CNN architectures were trained, they were evaluated based on a number of evaluation metrics (mentioned in the next section) to determine which model had a higher capacity for accuracy relative to the other models. Then, after this model was determined, it was tested on images with different situations to determine how it will respond and adopt to these variations. A range of thresholding approaches were carried out to investigate how well the model localized and segmented the images. The softmax classifier [32] was the foundation behind the classification process. It gives a binary type-based response (1s and 0s) as a first-hand segmented image and a probabilistic score (from 0 to 1) for the purpose of thresholding the segmented image.

### 2.3. Evaluation Process and Metrics

The performance of the proposed method was evaluated by comparing the pretrained models with different evaluation metrics. Before mentioning the evaluation metrics, it is important to explain the four classes of statistical classification, also called confusion matrix, which is not familiar in construction fields. These are mentioned as follows [33]:

- True positives (TP): an outcome where the model correctly predicts the positive class.
- True negative (TN): an outcome where the model correctly predicts the negative class.
- False positive (FP): an outcome where the model incorrectly indicates the positive class, but it is actually negative.
- False negatives (FN): an outcome where the model incorrectly indicates the negative class, but it is actually positive.

Based on the confusion matrix and other operations, in this work, four types of evaluation metrics were used to determine the capability and tendency of detection of the architectures. The quality of the learning algorithms is generally evaluated by analyzing the metrics [34].

The first one is the accuracy. It is the ratio of correctly classified pixels, regardless of class, to the total number of pixels. In simpler terms, it is the ratio of the sum of TP and TN to the sum of TP, TN, FP, and FN. Recall, also called sensitivity, is the second one, which for our case represents the percentage of correctly identified pixels for each class. It is the ratio of correctly classified pixels to the total number of pixels in that class, according to the ground truth. In simpler terms, it is the ratio of the TP to the sum of TP and FN. The main difference between accuracy and recall is that the accuracy does not consider the class, which makes it a generalized evaluation method compared to the recall. 

Intersection over union (IoU), sometimes called ‘Jaccard Index’, is another parameter used to evaluate the neural networks. It is a metric for measuring the overlap between the ground truth class and the predicted class. The lower the value, the more the prediction capability decreases. Finally, time, i.e., running time, is also a parameter for evaluating the efficiency of the networks.

## 3. Results and Discussion

### 3.1. Effects of Epoch and Batch Size

**Figure 3:**
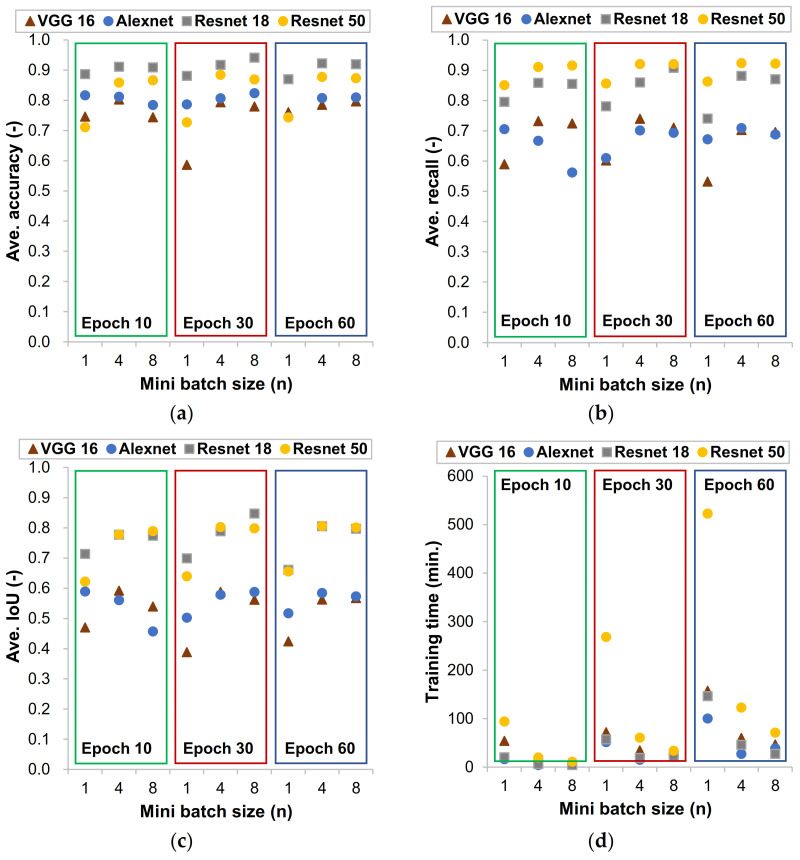
Evaluation of CNN models based on the evaluation metrics: averaged values of accuracy (**a**), recall (**b**), and IoU (**c**) for testing datasets and (**d**) training time for dataset.

The retrained models were evaluated based on the evaluation metrics proposed in the above segment by varying the epoch and the batch size. Figure 3 presents the evaluation metrics of the models with various epoch and batch sizes. It can be seen that the model with ResNet CNN architecture as a backbone have a higher score of accuracy, recall, and IoU than the other models on average. This was achieved despite the lesser number of parameters that DeepLab V3+ with ResNet backbone has compared to the others. The main reason behind this is that the ResNet series is a very deep residual network with a much lower number of parameters compared to the other two models. It has what we call a skip connection and features heavy batch normalization in which the output layer is the result of the function of the pervious layer and the input of the previous layer. This will increase the depth of the learning rate of each layer as it goes. It is usually assumed that stacking more layers will result in an increase of the accuracy of the network, but this is not the case. The accuracy accumulates and will go down rapidly because of the vanishing gradient problem. This means that as the layers are deeper and have more parameters, the initial layers will become insignificant during back propagation [35]. Therefore, this will cause the accuracy to drop rapidly. However, since the DeepLab V3+ model using ResNet series uses shortcut connections these gaps will not be created. This makes this model very efficient and effective for our objective.

**Figure 4:**
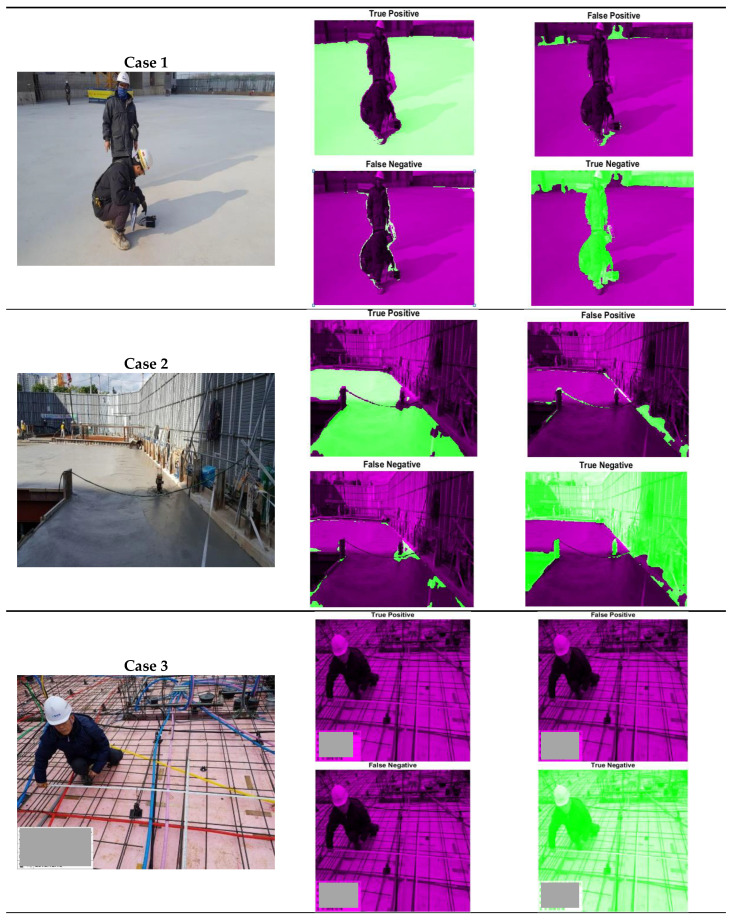

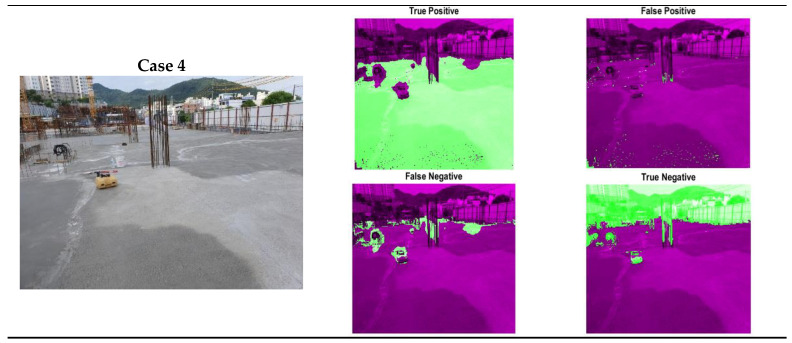
Examples of true positive (TP), true negative (TN), false positive (FP), and false negative (FN) from images (bright green: classified area for each selection criteria).

On the other hand, compared to the ResNet 18 model, the ResNet 50 required much more training time as shown in Figure 4, while the averaged accuracy and recall values were seldom improved. Therefore, in this study, the DeepLab V3+ model using a ResNet 18 backbone was selected for further works.

The other interesting thing was, as the batch size values increase maintaining the epoch values constant, the predictability of the model was increased. However, this does not mean that increasing the batch size will always have a good implication. The CNN models were trained in a stochastic gradient decent optimization method (SGDM). This method is based on updating the weights of the parameters to minimize the loss function until it converges to the minimum value. Therefore, with a smaller batch size, the parameters will be updated more. Unfortunately, a batch size more than eight was not possible in the test environment due to the GPU’s limitations. Even though we used the batch normalization (BN) parameter in the CNN models, the BN itself was bounded by the mini batch size chosen by the training options. Therefore, the option of mini batch size was crucial since it was the basepoint for other parameters. From the investigation done on the evaluation metrics, the model that was chosen was trained with an epoch of 30 and a batch size of 8 for our further analyses.

### 3.2. Case Study: Thresholding Approaches

The averaged accuracy and recall of the pretrained models may be enhanced as the number of datasets being trained increases. However, in the case of images with very diverse objects and complex situations such as those of construction sites, the important thing in pixel-level segmentation is not only the overall and averaged accuracy of the dataset but also local accuracy for each single image.

Figure 4 is the visual representations of TP, TN, FP, and FN predicted by the ResNet 18 model. As is well known, many of the objects in the images from the construction sites were gray. For this reason, brightness spectra threshold techniques were hard to use for judging the area covered by concrete in general. Moreover, there were many limitations in clearly classifying the fresh, young, and old concrete with similar colors. As shown in Figure 4, the area of TP from the pretrained models shows relatively good agreement with of actual area of concrete. In Case 1 in Figure 4, there are concrete slabs in the center and concrete walls of different ages in the back, and there is also a formwork in the distance. For this image, the main region of interest was the concrete slab in the center, so that only it was labeled as concrete, and the most of TP area from the model agreed with this. However, there was some FP area at the boundary between the area of people and concrete. At the same time, concrete walls at the back that were labeled as nonconcrete were predicted as concrete. This area was judged as FP, but it was concrete although it was out of the ROI. A similar phenomenon appeared in Case 2 as the FP at the edge of the concrete. Some range of the area of FP in Case 2 was also the concrete party wall cast before pouring fresh concrete. In other words, although it could not distinguish between concrete of different ages as a professional would, this model could segment the overall concrete section.

In Case 3 of Figure 4, there are some objects (actually buried pipes) similar in color to concrete in the back part of the image, but as a result, all parts were predicted as not concrete. In this case, the TP value became 0. In Case 4, the surface of the young concrete slab had different colors. It should be mentioned that, in practical, the plastic sheets were covered on the fresh concrete after pouring and finishing to prevent the plastic shrinkage, and there were cases where the sheets were contacted to the surface of the concrete depending on the area. The surface color and texture of the young concrete may have been changed by this contact as it affects the drying and bleeding condition. But the model predicted the TP and TN well with minor mis detections.

**Figure 5:**
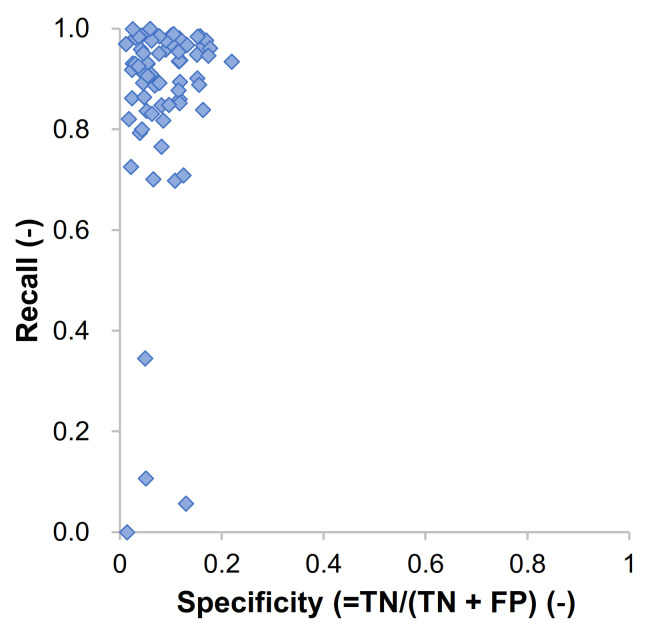
A receiver operating characteristic (ROC) of randomly selected test dataset.

**Figure 6:**
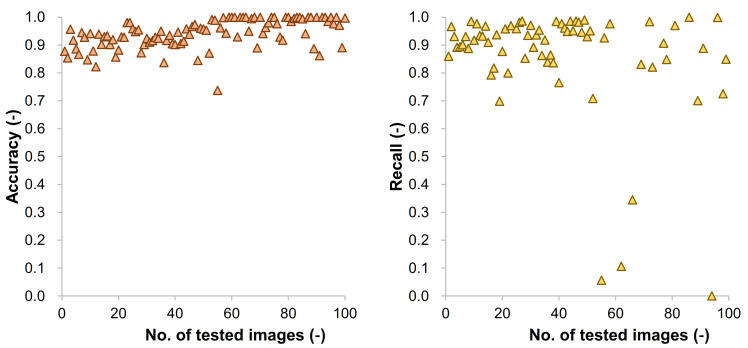
Accuracy and recall values of the randomly selected test dataset.

Using the number of pixels of TP, TN, FP, and FN, the values of the evaluation metrics were calculated for each image in the dataset. Figure 5 presents the relationship between the recall and specificity (= TN/(TN + FP)) for 100 randomly selected images from testing datasets. This type of figure is called the receiver operating characteristic (ROC) plot, and it presents the performance of a classification model. As mentioned above, the recall, also called the true positive rate (TPR), is the proportion of positive instances classified correctly, for this case, how the model classified the concrete areas suitably. Meanwhile, sensitivity, also called the true negative rate (TNR), is the portion of not concrete areas that are correctly classified. Most of the results were close to the point of recall = 1 and specificity = 0, which meant that the proportion of TP was high in general while that of TN was low. Thus, from Figure 5 we can infer that the credibility of the model was quite high. Figure 6 shows the results of accuracy and recall for 100 randomly selected images from testing datasets. The accuracy, which contained both TN and TP, was within a range of 0.9 ± 0.1 in most cases. Meanwhile, as shown in Case 3 of Figure 4, there was a case where the pixel numbers of TP were low as there was limited concrete area. If the model could not predict this limited area of concrete, i.e., FN for this case, then the recall values became very small as shown in the right side of Figure 6.

The prediction results of the model could also be obtained through a thresholding approach. As shown in Figure 2, the softmax classifier yielded both binarized (0s and 1s) and probabilistic values for each pixel after calculation. As is well known, the probabilistic value from the softmax function is a kind of hypothetical value from mathematical normalization. Therefore, the probabilistic values for pixel-level segmentation should be also evaluated for each image in both quantitative and qualitative ways.

**Figure 7:**
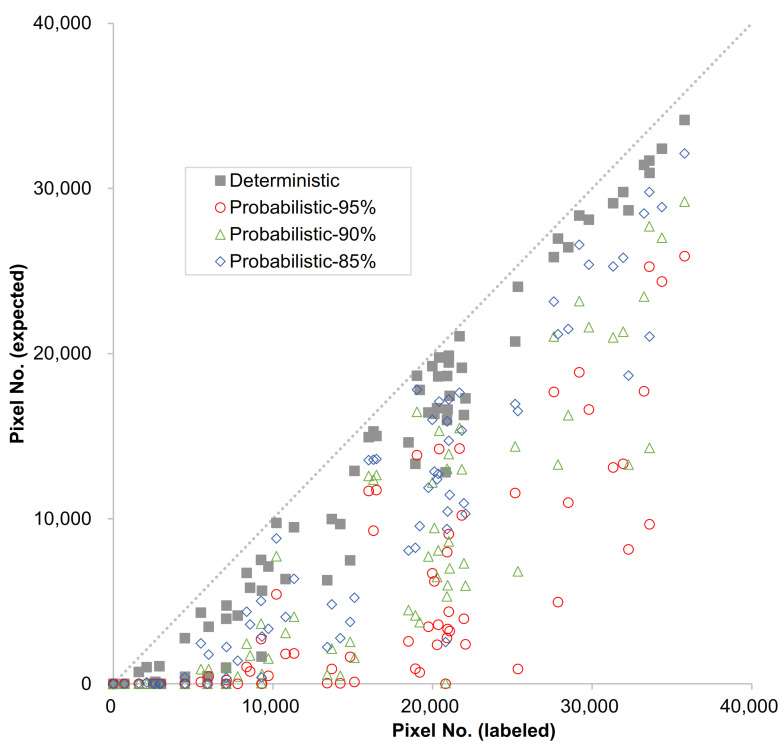
Labeled vs. expected pixel numbers of concrete area in images for testing set.

Figure 7 shows the comparison of the pixel numbers of the concrete area in a labeled image with those in prediction for 100 images randomly selected among the testing datasets. In ideal terms, both values may be identical if the model was perfectly trained. In the present study, the threshold value for probabilistic scores for the pixels was divided into three levels of 85%, 90%, and 95%. Figure 7 indicates that, as the threshold value increased, that is, the more stringent the criterion, the number of pixels classified as concrete decreased. Moreover, the number of predicted pixels in the case of the thresholding approach was less than those from the binarized results. The pixel numbers of concrete from binarized segmentation were in a somewhat similar range to those in the labeled images. It should be noted the higher range of thresholding values were the subset of the 50% thresholding method with a more refined filtering threshold.

**Figure 8:**
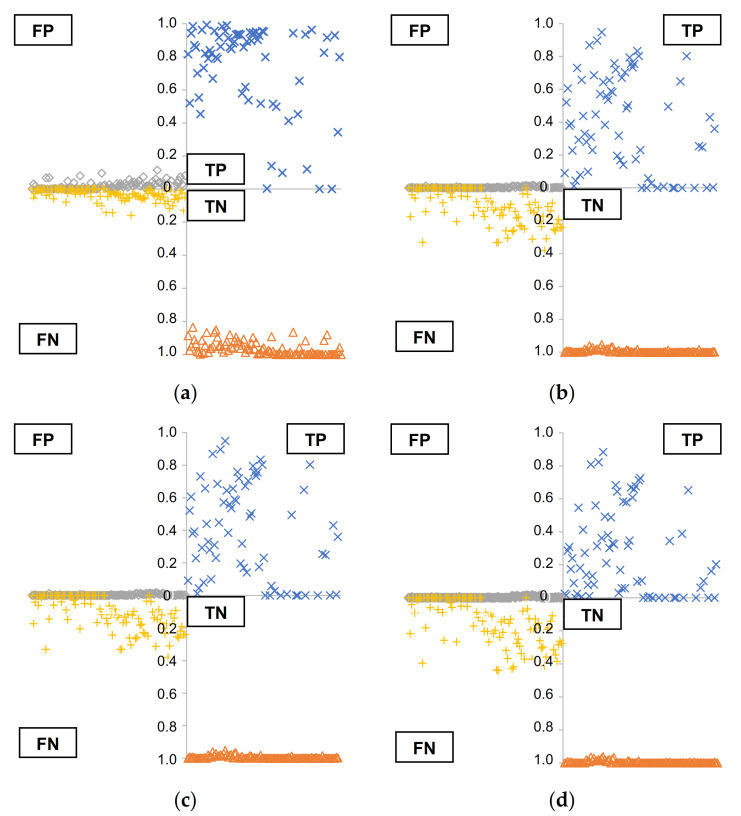
Confusion matrices of the model: (thresholding with 50% and above) (**a**) and with thresholding approaches of 80 % (**b**), 90% (**c**), and 95% (**d**) (x and y axes indicate sample image number of test dataset and percentages of each matrix, respectively).

Figure 8 shows the confusion matrices of the models for both approaches. In the case of the 50% thresholding, the values of TP were relatively higher than in other cases, which corresponded with the results in Figure 7. The values of FP for this case were also relatively higher within the range from 0 and 0.2, while the FN values were smaller. In the strictest case of 95% threshold, some of TP values were closed to 0, while most of the TN and FP values were 1.0 and 0, respectively. These values indicated that, through this approach, the model could predict the area covered by concrete well, but there was a possibility that some parts that were not concrete in actual were incorrectly predicted as concrete. On the other hand, when the criterion became stricter, some of actual concrete area could not classified as concrete, but at the same time, the incorrect prediction as concrete became almost zero. Therefore, the value of the threshold can vary depending on how to use this prediction model.

**Figure 9:**
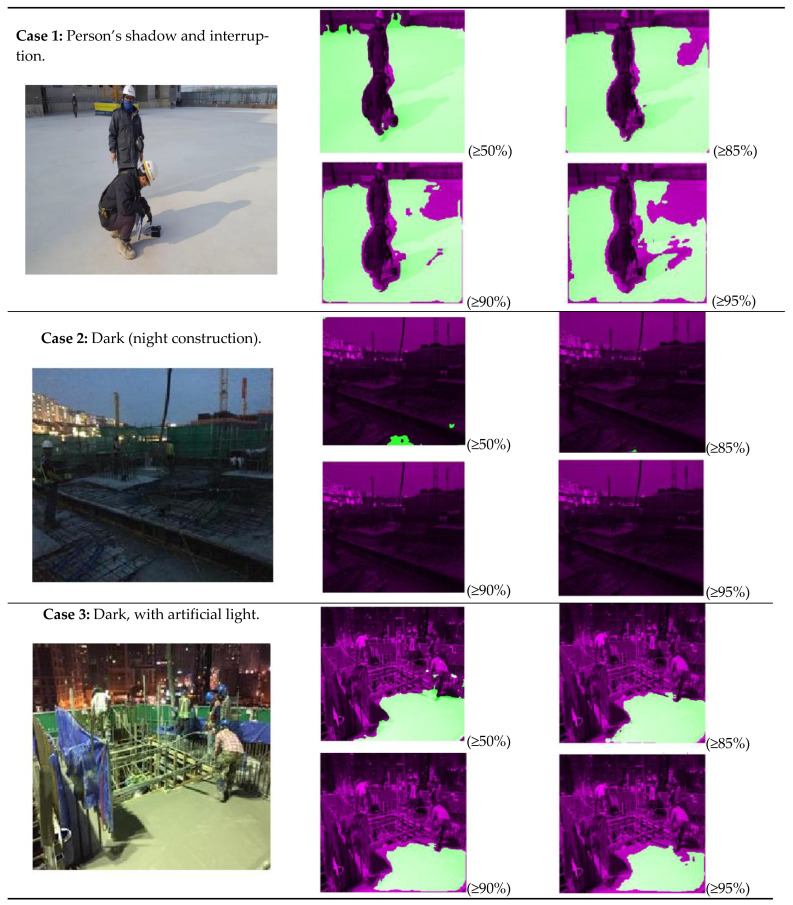

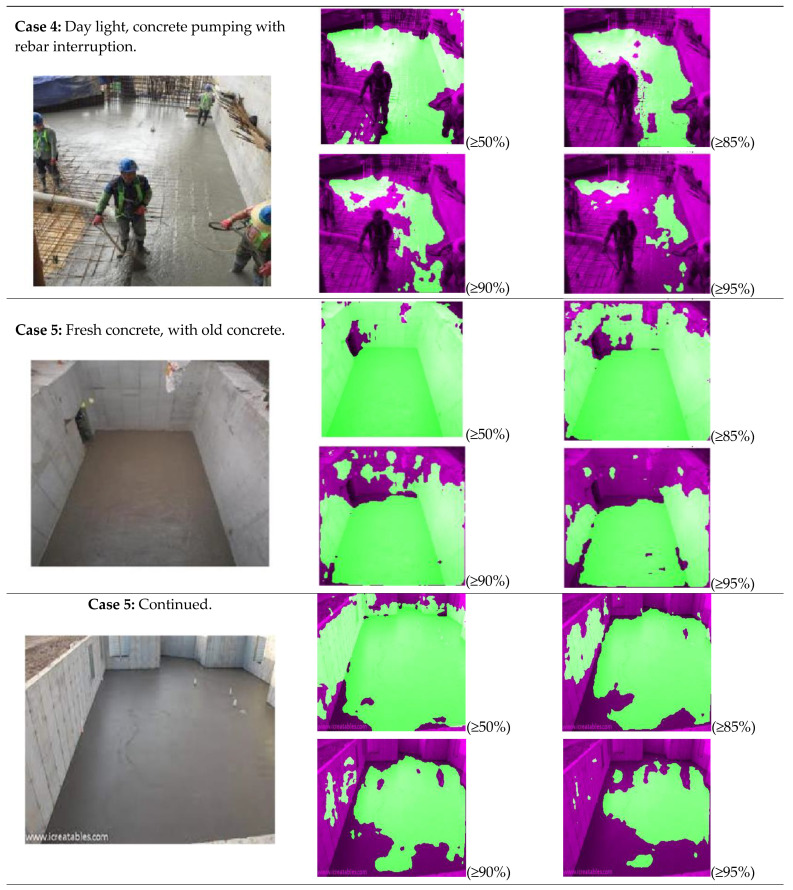

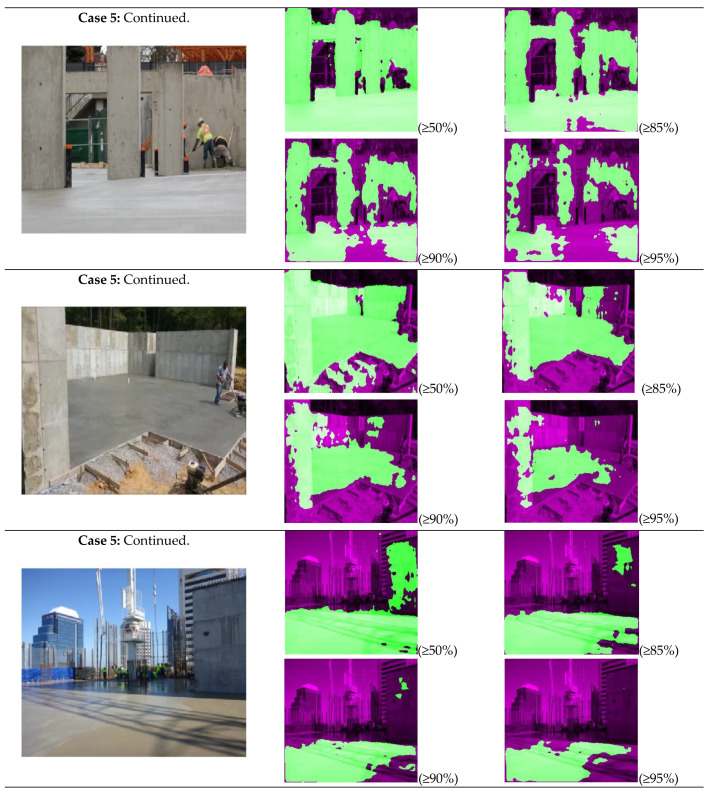

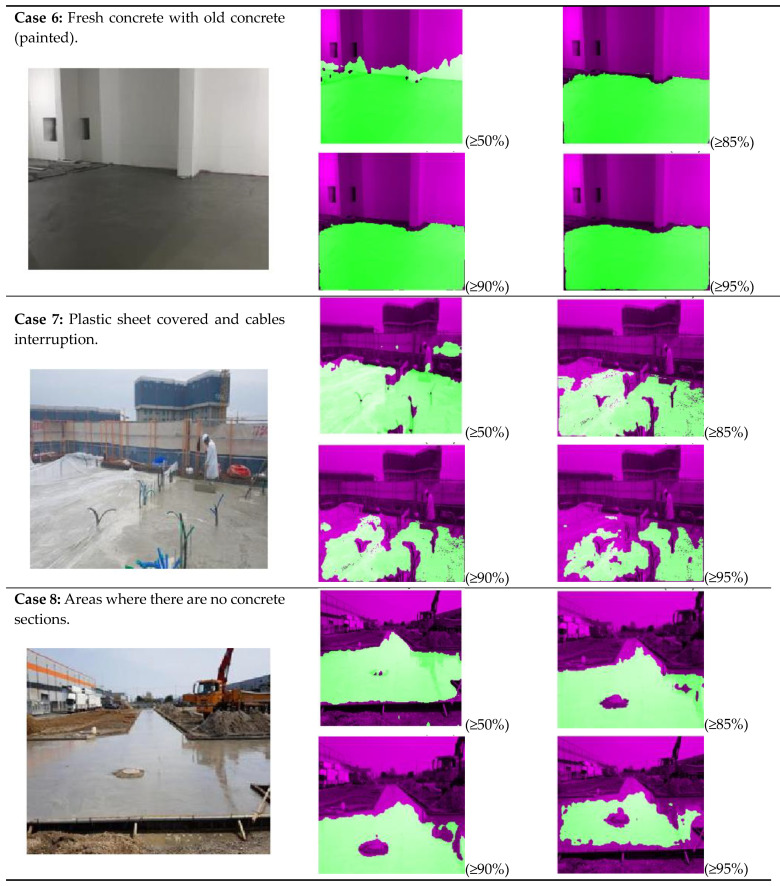

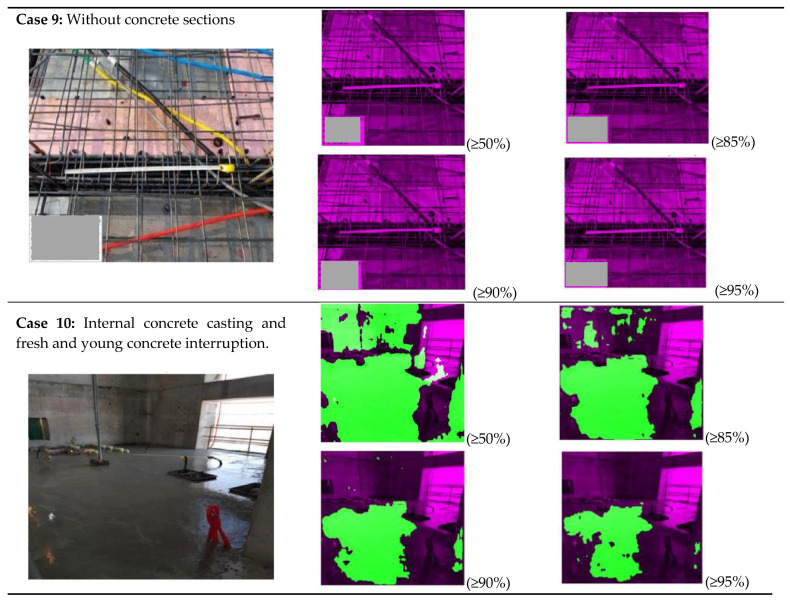
Examples of segmented images from the thresholding approaches (bright green: concrete; deep purple: nonconcrete).

Figure 9 shows the cases of the concrete area classified from the range of thresholding approaches. In Case 1 in Figure 9, as mentioned above in Figure 4, concrete walls of backside of the images, which were labeled as nonconcrete, were judged as concrete in the 50% thresholding approach, whereas they were not in the higher range thresholding values. By increasing the threshold values, the concrete area with a persons’ shadow became classified as nonconcrete. In Case 2, despite the very dark conditions, there was an actual concrete placement at the bottom of the image, and it was judged as concrete in the 50% thresholding segmentation. However, as the threshold values increased, this part was excluded.

In Case 3 of Figure 9, there were very dark shadows over the concrete because of strong artificial light. The concrete in this shadow could not be included in prediction area by any approaches in this work. There was some incorrect judgement in this image, i.e., the concrete stains on the worker’s clothes. This part could be excluded by an increase in the threshold values. In Case 4, the prediction area by the 50% thresholding approach included a vertical wall that was actually concrete but was out of the ROI. However, when it was necessary to focus only with fresh concrete, it was also necessary to increase the threshold value as well.

In Case 5 of Figure 9, five examples were provided to see whether the model can distinguish between fresh and old concrete. Based on the results, it was impossible to distinguish between the two using any of the approaches. This was mostly attributed to the reason that both have comparable characteristics and patterns, particularly in color and texture. As a result, further research is required to resolve this issue. Moreover, additional classification method such as the convex hull concept may be helpful for more detail segmentation. In Case 6 of Figure 9, the original image contained a painted concrete wall while the ROI was young concrete at the slab. In the 50% thresholding approach, some of the painted wall was included in the prediction area, it could be excluded by applying higher threshold values.

Case 7 in Figure 9 shows the site of concrete curing where a plastic sheet was protecting the fresh or young concrete. As mentioned in Figure 1, the areas where the concrete directly attached with the plastic sheet were labeled as concrete. As a result of the binarized approach, not only was the section where the plastic sheet was not attached with concrete judged as concrete but also some area of the temporary wall to prevent the wind was incorrectly evaluated as concrete, which are FPs. This problem was sufficiently eliminated when the threshold value was increased. When the threshold was over 90%, the floating part of the plastic sheet was removed. Case 8 in Figure 9 presents the concrete slab directly after pouring, which directly mirrors the surrounding environment due to bleeding of water. A heavy equipment was reflected in some portion of the image. This reflected portion was ignored in the 50% thresholding segmentation. However, the manhole in the middle in concrete was included at the same time. When the threshold value was increased, the manhole part was removed and the reflected part of the image was excluded and labeled as not concrete. 

In Case 9 of Figure 9, the original image contained a plate with a color similar to that of concrete, which was the metal forms, and this area was labeled as nonconcrete. As a result of prediction, it was confirmed that all parts were nonconcrete, regardless of the threshold value. Case 10 shows a complex situation in which newly poured fresh concrete and young concrete existed at the same time. The natural light was strong, and bleeding occurred in the newly poured concrete, so that there were reflections and dark shadows. In this case, it was impossible to separate the two types of concrete through the binarized approach and also hard to contain the concrete area covered with the shadow and reflection. As the threshold value was gradually increased, only the floor, which was fresh concrete, was classified as concrete. However, it was difficult to include the concrete area with shadows and light reflections.

In general, the cases listed above were intended to show how well the model would perform in real-life circumstances. It is clear from the findings that the model is capable of segmenting concrete areas, whether fresh or old, from images of construction sites. Additional adjustments can be made to improve detection accuracy by integrating the model with other techniques and refining the approaches to data labeling and training. This allows for the development of a more accurate and reliable concrete area segmentation model.

## 4. Conclusions

Deep-learning-based image segmentation is highly applicable in the construction industry especially for monitoring and inspection purposes. The main target of this investigation was to identify the area covered by fresh and young concrete from images of construction sites so that it can be used for various applications. Dataset for training contained about a thousand RGB images taken from actual construction sites in South Korea. A pixel-level labeling of concrete areas was conducted for the dataset, and various CNN architectures, including AlexNet and SegNet, were applied to train this. The performance of these deep-learning models for the prediction of concrete area was analyzed by means of quantitative and qualitative methods.

The DeepLab V3+ using the ResNet 18 model with a training condition of an 30 epoch and mini batch size of 8 gave the best performance among the models in terms of accuracy, recall, and training time. With this model, the averaged accuracy and recall for a randomly selected test set were higher than 0.9 and 0.87, respectively. It should be emphasized that this model could segment the concrete area overall, although it could not judge the difference between concrete of different ages as a professional would. Moreover, by adjusting the threshold values for the softmax classifier, the incorrect prediction (false positive) as concrete became almost zero. The present model worked correctly when a single type of concrete area was contained in the images. In this case, the other objects frequently included in the images of a construction site, such as person (worker), concrete forms, pipes and lines, and metallic or plastic sheet and panels, were successfully excluded from the prediction area for concrete. When a strong reflected light or shadow were on the concrete, these areas sometimes could not be classified as concrete.

To summarize the aforementioned facts, it was critical to prepare the visible conditions or situations that target concrete so that it could be properly categorized in order to successfully apply these deep-learning-based segmentation approaches. In some of the images that were taken in a darker light, there was some misidentification of the area by the model. However, overall, this problem was minimized when the threshold limit was increased even though some areas that were covered by concrete were not recognized. Furthermore, the FP and FN values of the model were lower, implying that the model effectively identified nonconcrete regions, which is an advantage considering that detecting nonconcrete areas is also essential.

## Figures and Tables

**Table 1:** Summary of the pretrained CNN models used.

CNN Model	FCN	SegNet	DeepLab V3+	DeepLab V3+
Backbone encoder	AlexNet [9]	VGG 16 [28]	ResNet 18 [16]	ResNet 50 [16]
Parameters	~60 million	~138 million	~11 million	~23 million
Neurons	650,000	5504	4096	
Input image size	227 × 227	224 × 224	224 × 224	224 × 224
Convolutional layer	5	3	5	5
Fully connected layer	3	3	1	1
Max pooling layer	5	5	2 (1 average)	2 (1 average)
Total connections	24	40	78	192
Total layers	25	41	71	177
Output type	Classification	Classification	Classification	Classification

**Table 2:** Training parameters used in the training process of the dataset.

Training Parameters	Options Used
Optimization algorithm	SGDM
Momentum	0.9
Execution environment	Single GPU
L2 regularization	0.0005
Shuffle	Every epoch
Initial learn rate	0.003
Learn rate schedule	Piece-wise
Mini batch size	Varied
Max epochs	Varied

**Table 3:** Machine and software environment implemented in the training process.

Machine and Software Environment	Selections Used
System type	Microsoft Windows 10, 64-bit
RAM	24 GB
Processor	Intel(R) Core™ i7-7700 CPU @ 3.6 GHz
Graphics driver	NVIDIA GeForce RTX 2080 Ti 11 GB
Execution environment	MATLAB, R2020a

## Data Availability

The data presented in this study are available on request from the corresponding author.
